# A Dynamic Energy Budget Model for the Non-Continuous and Biphasic Growth of the Pond-Cultured Swimming Crab, *Portunus trituberculatus*

**DOI:** 10.3390/biology14121682

**Published:** 2025-11-26

**Authors:** Yi Jiang, Fan Lin, Jingyan Zhang, Ming Bao, Baoquan Gao, Jitao Li, Xianliang Meng

**Affiliations:** 1 State Key Laboratory of Mariculture Biobreeding and Sustainable Goods, Yellow Sea Fisheries Research Institute, Chinese Academy of Fishery Sciences, Qingdao 266071, China; 2 Laboratory for Marine Fisheries Science and Food Production Processes, Qingdao Marine Science and Technology Center, Qingdao 266237, China; 3 Key Laboratory of Aquatic Genomics, Ministry of Agriculture and Rural Affairs, Yellow Sea Fisheries Research Institute, Chinese Academy of Fishery Sciences, Qingdao 266071, China

**Keywords:** *Portunus trituberculatus*, crab, molt, pond aquaculture, dynamic energy budget, piecewise growth

## Abstract

The swimming crab, *Portunus trituberculatus*, is an important mariculture crustacean species in China. To quantitatively characterize its discontinuous and biphasic growth under intensive aquaculture conditions, we developed a modified growth model based on the dynamic energy budget (DEB) theory. The model incorporates a discontinuous growth representation that explicitly accounts for the molting process. Molting events were parameterized using a threshold ratio (α), defined as the carbon weight-to-wet weight ratio, with stage-dependent critical values derived from laboratory experiments and published datasets. Laboratory experiments were designed to estimate the energy-budget-related parameters together with data from the literature. In addition, by introducing a feedback mechanism for the energy demand of ovarian development, a set of κ values was calibrated with data from the literature to reproduce the stage-specific growth determined by ovarian development. The model was validated with growth data from several published studies related to the growth of *P. trituberculatus*, as well as measurements from the aquaculture ponds. Multiple goodness-of-fit indices (R-square, modeling efficiency, and Theil’s inequality coefficient) confirmed that the modified DEB model accurately reproduced both wet weight and ovarian growth trajectories, along with the characteristic non-continuous pattern of molting-driven wet weight increase. The individual growth model can be a useful tool for optimizing the intensive culture practice of the swimming crab in the aquaculture ponds and assessing the environmental impact of crab aquaculture.

## Simple Summary

The growth of many decapods is characterized by discontinuous patterns driven by molting, as well as stage-specific shifts in energy allocation, particularly concerning gonadal development, which limits the applicability of conventional growth models. This study establishes a non-continuous and biphasic dynamic energy budget (DEB) framework for the swimming crab *Portunus trituberculatus*, an important aquaculture species, to link energy acquisition, molting, and reproductive development under culture conditions. By incorporating an α-based molting threshold and stage-specific energy allocation, the model quantitatively reproduces the observed staged growth and ovarian maturation processes. This framework offers a generalizable approach for describing molt-dependent energetics and supports effective management of crustacean aquaculture.

## 1. Introduction

The swimming crab, *Portunus trituberculatus*, widely distributed in the coastal waters of Asian-Pacific countries, is an important aquaculture species in China. In 2023, the production of *P. trituberculatus* reached 101,000 tons in China and constituted the majority of domestic swimming crab production [1]. As the market demand has continued to rise in recent years, the farming of this species has been moving toward intensive pond-culture models [2]. In current pond-culture practice, the growth of swimming crab is constrained by regional environmental conditions and seasonal temperature variation. Moreover, intensive aquaculture increases the burden on water-quality maintenance and the concern for environmental pollution. Therefore, there is an urgent need to establish a quantitative modeling framework that can predict the growth trajectories of crab, improve the production efficiency, and minimize the environmental impacts.

Individual-based models have become essential tools for aquaculture management, providing mechanistic insights into how energy assimilation, growth, and reproduction respond to environmental factors [3,4]. Among these, the dynamic energy budget (DEB) theory offers a universal physiological framework that links energy acquisition to metabolic processes throughout an organism’s life [5,6]. The DEB approach has been successfully applied to various aquaculture species, such as the anchovy, *Engraulis encrasicolus* [7]; the yellow croaker, *Larimichthys polyactis* [8]; the Pacific oyster, *Crassostrea gigas* [9]; the Yesso scallop *Patinopecten yessoensis* [10], the Chinese shrimp *Fenneropenaeus chinensis* [11], and the sea cucumber, *Apostichopus japonicus* [12]. These models have effectively improved the aquaculture management for these species.

However, decapod crustaceans exhibit distinct discontinuous growth driven by molting, which cannot be represented by conventional DEB formulations assuming continuous size change. Each molt results in a sudden increase in wet weight following carapace replacement and water uptake, whereas carbon biomass accumulates gradually between molts. To address similar discontinuous dynamics, Talbot et al. proposed a molting-trigger DEB model for the harbor crab, *Liocarcinus depurator*, where molting occurs once the carbon-to-wet-weight ratio (α) exceeds a threshold [13]. This concept provides a promising framework for modeling crustaceans with stepwise growth patterns.

In most DEB models, the ratio (κ) of the energy allocated to somatic maintenance and growth is fixed during an individual’s life course. However, the growth of many decapods exhibits a stage-specific pattern, and κ obviously is not fixed at different life stages. For *P. trituberculatus*, the development cycle of gonads is dependent on the stage of molts. Before the puberty molt (the last molt of the growth cycle), the swimming crab grows by cyclical molt, and its gonads develop very slowly. After the puberty molt, ovarian development accelerates sharply, diverting substantial energy toward reproduction [14]. The commonly fixed allocation parameter (κ) used in standard DEB models thus fails to capture this physiological shift. However, there has been no DEB model describing the stage-specific growth pattern in the swimming crab and other decapods.

The primary objective of this study is to develop and parameterize an individual-based DEB model for *P. trituberculatus* that captures its stage-specific, discontinuous growth pattern and ovarian development. The study introduces (i) a molting-trigger mechanism based on the carbon-to-wet-weight ratio (α), (ii) a segmented κ-rule reflecting stage-dependent energy allocation, and (iii) a feedback-controlled representation of gonadal energy demand. This framework provides a quantitative foundation for optimizing growth prediction and reproduction manipulation in intensive swimming crab aquaculture and could be useful for improving the aquaculture management and minimizing the environmental impact.

## 2. Materials and Methods

### 2.1. Model Structure and Formulation

#### 2.1.1. Conceptual Framework

**Figure 1:**
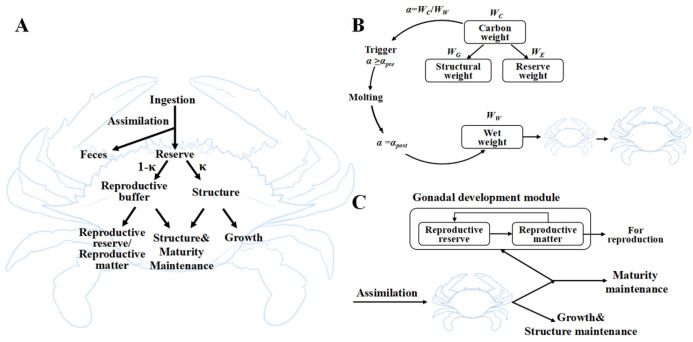
(**A**) Concept diagram of the standard DEB model for *P. trituberculatus*. In this model, all kappa fluxes (κ) refer to the maintenance and growth of somatic cells. (**B**) Schematic diagram of the α-threshold molting trigger mechanism. (**C**) Demand-driven gonad-loading feedback module activated since stage VII.

We developed an individual-based model grounded in the dynamic energy budget (DEB) theory to represent the bioenergetics of the swimming crab, *Portunus trituberculatus*. The concept diagram of the standard DEB growth model for *P. trituberculatus* is shown in Figure 1A. In the DEB framework, assimilated energy first enters a reserve compartment (E) and is then mobilized and allocated according to the κ-rule: a fraction κ supports somatic maintenance and structural growth, while the complementary fraction (1 − κ) fuels reproductive investment. To accommodate the discontinuous growth inherent to crustacean molting, the standard DEB structure was extended with an explicit molting trigger and an ovarian development module (std+ extension) [15]. The model state variables, fluxes, and governing equations are summarized in Supplementary Material Table S1.

#### 2.1.2. Molting Trigger via α-Threshold

Crab body wet weight ($W_{w}$) increases in a stepwise fashion at each molt due to the formation of a larger carapace and associated water uptake, while carbon weight ($W_{C}$) accumulates continuously between molts. We define an internal molting indicator, α, as the ratio $W_{C}$/$W_{w}$. When α exceeds a pre-molt value ($\alpha_{pre}$), a molting event is triggered; $W_{w}$ then jumps to a larger value, and α is reset to a lower, post-molt value ($\alpha_{post}$). In our implementation, $W_{C}$ is the sum of structural weight ($W_{G}$) and reserve weight ($W_{E}$). Between molts, $W_{C}$ increases via assimilation and growth while $W_{w}$ remains nearly constant, causing α to rise toward α_pre_; after each molt, $W_{w}$ increases sharply, and α is reset to $\alpha_{post}$ before beginning a new cycle. Figure 1B provides a schematic overview of the molting trigger mechanism, which is governed by an α-threshold.

#### 2.1.3. Ovarian Development and Segmented κ

Female *P. trituberculatus* exhibits a distinct shift in energy allocation at reproductive maturity: prior to the puberty molt, gonadal development is minimal, and energy is devoted to somatic growth; after the puberty molt, ovarian maturation accelerates and diverts a larger share of mobilized energy. We explicitly quantify ovarian development using a demand-driven feedback mechanism (DEB std+), which is illustrated schematically in Figure 1C. In this module, the conversion of reproductive reserves into ovarian tissue depends on reserve density, reproductive material density, their difference from a maximum density, and a growth coefficient. Densities are defined as carbon-weight ratios relative to structural weight. Because male reproductive investment is negligible (gonadosomatic index ≈ 0.2%) [16], we model only female ovaries. To reflect stage-dependent allocation, κ is segmented as follows: κ = 1.0 before stage VII (no ovarian investment), κ = 0.9 for stages VIII-X (slow ovarian development), and κ = 0.2 after stage X (rapid ovarian growth) [14].

### 2.2. Laboratory Experiments

To parameterize the DEB model, a suite of physiological experiments was conducted using the swimming crabs collected from commercial culture ponds in Weifang, Shandong Province (36°42′10.44″ N, 119°03′14.4″ E). All trials were carried out in a recirculating seawater system comprising 30 tanks (20 L each), maintained under stable conditions of 25 ± 1 °C, 30 ± 0.6 salinity, pH 7.6 ± 0.2, ammonia-N < 0.03 mg L^−1^, and dissolved oxygen at 6.9 ± 0.2 mg L^−1^. Crabs were acclimated for seven days before experimentation and fed fresh fish daily. Detailed protocols and methods are described in the Supplementary Material S2.

#### 2.2.1. Oxygen Consumption and Arrhenius Temperature (TA)

To determine the Arrhenius temperature dependence of the metabolic processes, oxygen consumption rates (ROCs) were measured at five experimental temperatures (7, 13, 19, 25, and 31 °C). Five size classes were examined, combining newly obtained data for larger crabs (100–175 g $W_{w}$) with published datasets for smaller individuals [17,18]. Crabs were gradually acclimated to each temperature at a rate of approximately 1 °C per day and maintained at the target temperature for 3 days before measurements. ROC was determined using the hydrostatic respirometry method [19]. TA can be estimated from the linear regression between the Napierian logarithm of ROC and the reciprocal of the absolute temperature, following Van der Meer [20].

#### 2.2.2. Starvation Experiment

To characterize reserve utilization and maintenance metabolism, 36 crabs (125.7 ± 32.0 g $W_{w}$) with low gonadal development were subjected to a starvation trial at 25 °C. Six individuals were sampled every two days to determine $W_{w}$, carbon weight ($W_{C}$), and ROC. $W_{w}$ was recorded after blotting surface water, while $W_{C}$ was obtained by drying and ashing body tissue. Energy content was measured using a PARR 1281 bomb calorimeter (PARR Instrument, Moline, IL, USA). ROC was measured as described above. These determination results were later used to estimate structural maintenance rates ($\langle \dot{P_{M}}\rangle$), maximum reserve density ($\langle E_{m}\rangle$), energy content of reserves ($\mu_{E}$), and weight-specific costs for structure ($\langle E_{G}\rangle$) in the DEB model, following procedures described by Ren and Schiel [21].

#### 2.2.3. Feeding Experiment

To estimate assimilation efficiency (AE) and the potential maximum assimilation rate ($\left\{{\dot{p}}_{Am}\right\}$), eight crabs (33.5 ± 27.2 g $W_{w}$) were maintained individually in 20 L tanks under ad libitum feeding conditions. After a 24 h fasting period to clear gut contents, each crab was provided with excess fresh fish. Three control tanks without crabs were used to quantify feed loss due to dissolution. After feeding, all uneaten feed and feces were collected, dried, and analyzed for energy content with the PARR 1281 calorimeter. AE was computed as the proportion of ingested energy retained after subtracting fecal losses, and $\left\{{\dot{p}}_{Am}\right\}$ was later derived by scaling assimilation to the structural weight of individuals.

#### 2.2.4. Ovarian Energy Budget

The energetic investment associated with ovarian development was assessed using female crabs collected at two maturity stages: before and after the puberty molt. Ovaries were carefully dissected, dried, and weighed, and the energy contents of both ovarian and somatic tissues were determined by PARR1281 oxygen bomb calorimeter. Carbon composition was analyzed by combustion, and these measurements were later used to calculate the energetic cost per unit ovarian tissue ($\langle E_{B(A)O}\rangle$) and to estimate the maximum density of reproductive material ($m_{Gm}$) incorporated into the DEB framework.

#### 2.2.5. Molt Ratio Determination

Molting dynamics were parameterized using the ratio of body carbon weight to body wet weight (α = $W_{C}$/$W_{w}$) measured in females at different molt stages from June to October. Freshly molted crabs were weighed immediately after carapace hardening to determine the post-molt α ($\alpha_{post}$). The pre-molt α ($\alpha_{pre}$) was derived from paired pre-molt and post-molt measurements, combined with data reported by Gao et al. [22]. The resulting stage-specific α-thresholds were incorporated into the model as rules for initiating molting events.

### 2.3. Model Simulation and Evaluation

**Figure 2:**
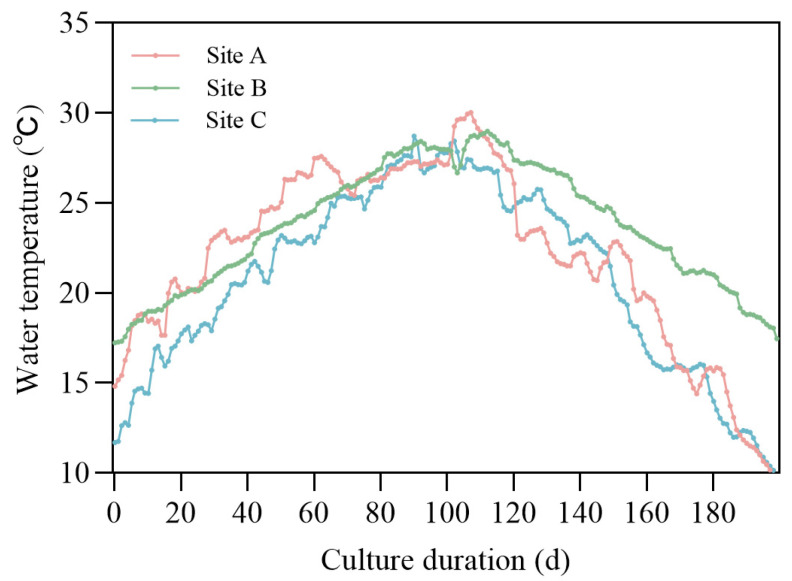
Temperature data used for driving the individual model at different geographical locations.

The DEB model equations in Supplementary Material Table S1 for *P. trituberculatus* were implemented with a finite difference method with Python 3.8.18 under the pond aquaculture scenario; the function response was assumed to be appropriate (*f* = 0.9) during the simulation. As in practical aquaculture environments, ponds are typically supplied with sufficient feed to meet the crabs’ needs. The driving temperature was the sea surface temperature near the aquaculture location and was downloaded from the online database provided by NOAA Optimum Interpolation 1/4 Degree Daily Sea Surface Temperature Analysis. The temperature data was interpolated to the model time series with a 1-day time step using a linear method (Figure 2).

Model validation used four growth datasets (Table 1) comprising data from the literature [23,24], previous observations from our laboratory, and an ovarian-mass dataset from previous studies [25]. The simulation lasted for 180 days, covering the culture period from May to October, from growth stage Ⅰ until the puberty molt of *P. trituberculatus*. We assume that the individuals in the dataset had just completed molting. We infer the initial growth stage through the initial $W_{w}$, thereby selecting an appropriate $\alpha_{pre}$ value for subsequent molting events, and calculate the initial $W_{C}$ based on the initial $W_{w}$ and $\alpha_{post}$.

We calibrated stage-specific κ values to reproduce the empirically observed shift in ovarian development. Calibration proceeded iteratively by comparing simulated ovarian trajectories against experimental data and adjusting κ within biologically plausible ranges until optimal agreement was achieved. Parameter sensitivity was evaluated following Majkowski [26], each major parameter was perturbed by ±10%, and the sensitivity index was computed as the relative change in predicted $W_{C}$ across n simulated days.

The performance of the DEB model for *P. trituberculatus* was evaluated by calculating the coefficient of determination (R^2^) for simulated and observed values [27], the model efficiency (ME), and Theil’s inequality coefficient (U). R^2^ = 1 denotes perfect correspondence; ME = 1 indicates predictions outperform the mean; U = 0 indicates ideal accuracy (definitions and formulae are provided in the manuscript). These statistics were applied to wet-weight and ovarian-mass time series at observed sampling dates. Further details of the goodness-of-fit evaluation and sensitivity analysis are available in Supplementary Materials S2.

## 3. Results

### 3.1. Experimental Results and Model Parameters

**Figure 3:**
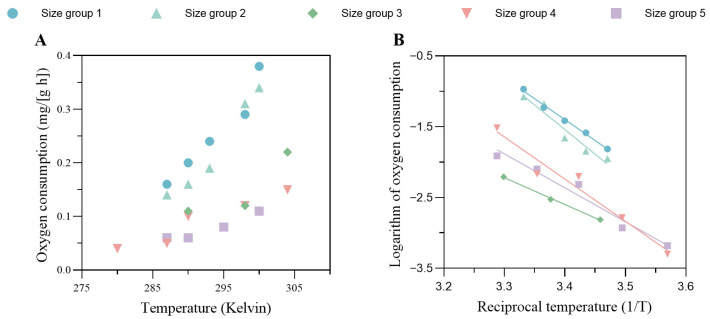
(**A**) The oxygen consumption per unit wet weight against the absolute temperature for different size groups. (**B**) Linear regression of the logarithm of oxygen consumption against the reciprocal temperature. These regression models are used to determine the Arrhenius temperature for the DEB model. The information on the size groups is shown in the legend. The body weights of each size group were as follows: size group 1, 1.17 ± 0.03 g; size group 2, 57.57 ± 1.16 g; size group 3, 78.19 ± 7.76 g; size group 4, 100.88 ± 11.22 g; size group 5, 174.97 ± 25.72 g.

The results from the oxygen consumption experiment are shown in Figure 3. Generally, the oxygen consumption per unit wet weight of the crab gradually increased with rising temperature, and smaller crabs had higher oxygen requirements, as shown in Figure 3A. The linear regression between the logarithmic value of the ROC and the inverse of water temperature (thermodynamic temperature, Kelvin) for the five size groups is shown in Figure 3B. The Arrhenius temperature, TA, for each size group was estimated as the absolute value of the slope from each linear regression, and the averaged value of the five datasets was taken as the Arrhenius temperature for the DEB model, which was 5482 ± 1214 K.

**Figure 4:**
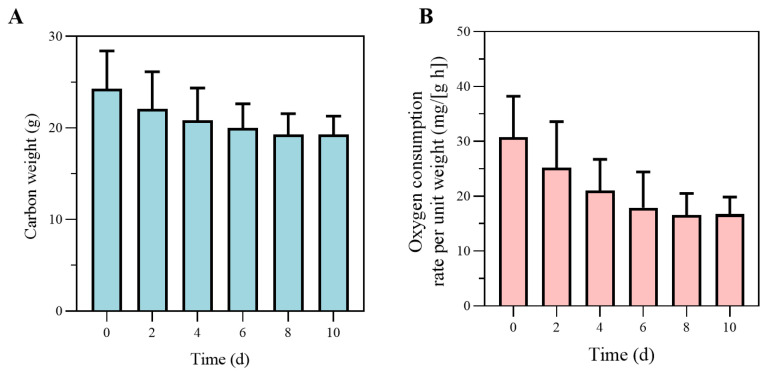
(**A**) Changes in carbon weight during the starvation experiment. In this context, carbon weight is contributed by structural weight and reserves, with no contribution from the ovaries. The stabilization of carbon weight indicates the depletion of energy reserves and a shift to a more stable metabolic state. (**B**) The changes in oxygen consumption rate per unit weight during the starvation experiment. The decrease and eventual stabilization of oxygen consumption reflect the crab’s metabolic adjustment to prolonged starvation.

**Figure 5:**
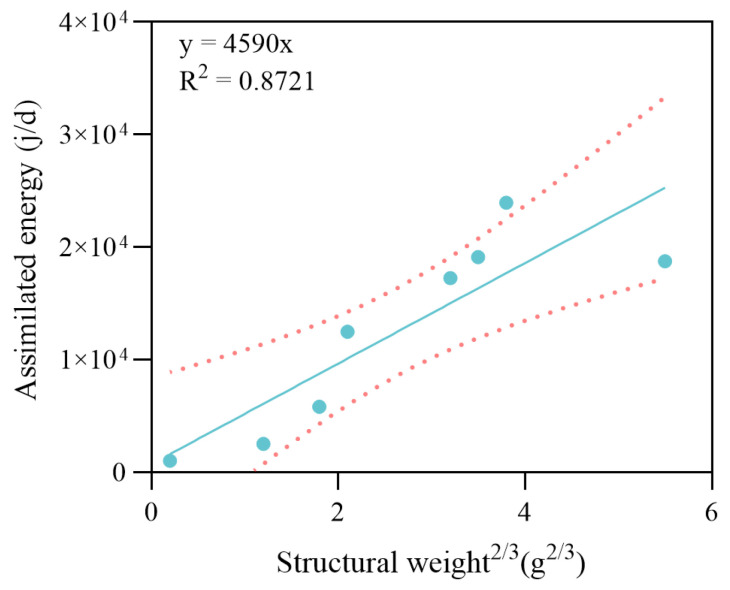
Linear regression analysis between structural weight^2/3^ and energy assimilation rate of the crabs. The dots represent the observed data points from the experiment. The solid line indicates the fitted linear regression, and the dotted lines represent the 95% confidence intervals.

The results for carbon weight during the starvation experiment are shown in Figure 4A. Throughout the experiment, $W_{C}$ gradually decreased and reached an approximately constant level after 8 d, which approached the structural weight as the reserves emptied. As shown in Figure 4B, ROC decreases rapidly after starvation, in line with the $W_{C}$ decrease, and stays almost constant after 8 d. Therefore, the carbon weight of the crab is assumed to be composed of sole structural weight after 8 d of the experiment, and $W_{C}$ approximately equals $W_{G}$ at this point. After starvation for long enough time, $W_{C}$ decreased by about 20%; the typical ratio of $W_{G}$ to $W_{C}$ for cultured *P. trituberculatus* was approximately β = 0.79 for the experimental crabs. The maximum reserve density ($\langle E_{m}\rangle$) was estimated as 3860 J·g^−1^. The energy content of reserves ($\mu_{E}$) was estimated as 18,750 J·g^−1^. Using an oxygen energy equivalent of 15,496 J·g^−1^, together with the terminal carbon weight, the structural weight specific maintenance rate ($\langle \dot{P_{M}}\rangle$) was 310 J·g^−1^·d^−1^. The weight-specific costs for structure ($\langle E_{G}\rangle$) were measured with the last group of crabs; with approximately constant carbon weight, the energy for specific structural growth was estimated to be 51,180 J·g^−1^. The results of the feeding experiment were used for assimilation parameters. The mean assimilation efficiency (AE) across size classes was 0.95. Figure 5 shows the scatter plot of the energy assimilation rate against the 2/3 power of the crab structural weight, which was used to scale the surface area.

**Figure 6:**
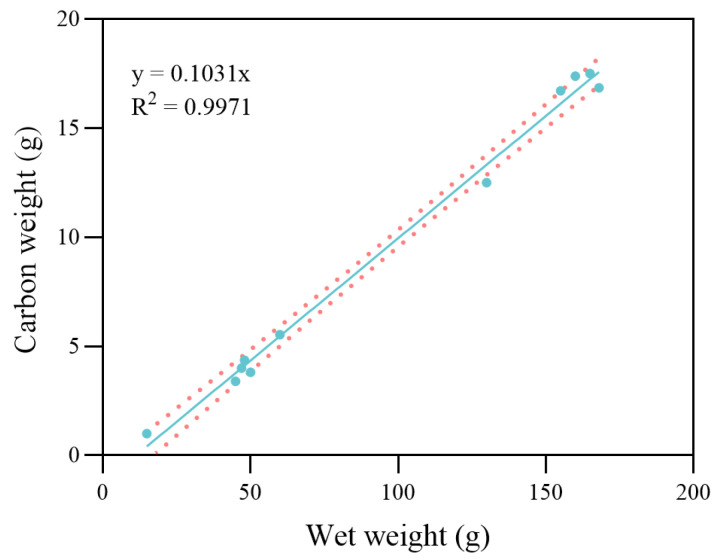
Linear regression analysis between wet weight and carbon weight in freshly molted crabs. The dots represent the observed data points from the experiment. The solid line indicates the fitted linear regression, and the dotted lines represent the 95% confidence intervals.

The structural weight of the maximum specific assimilation rate ($\left\{\dot{P_{Am}}\right\}$) was 4590 J·g^−2/3^·d^−1^. The energy cost per unit weight of gonad production was determined experimentally. The value for gonad production before the reproduction molt was $\langle E_{BO}\rangle$ = 36,690 J·g^−1^ for the slow developmental period, and the value after reproduction molt was $\langle E_{AO}\rangle$ = 66,200 J·g^−1^ during the rapid developmental period. A scatter plot of carbon weight against wet weight of all the freshly molted crabs is shown in Figure 6. According to the linear regression results, the $\alpha_{post}$ was approximately equal to 0.1 for all experiment crabs. The estimated range of $\alpha_{pre}$ was between 0.15 and 0.44 during different growth periods. The α values to trigger each molt for different stages are summarized in Table 2 according to the results.

### 3.2. Simulation Results and Sensitivity Analysis

**Figure 7:**
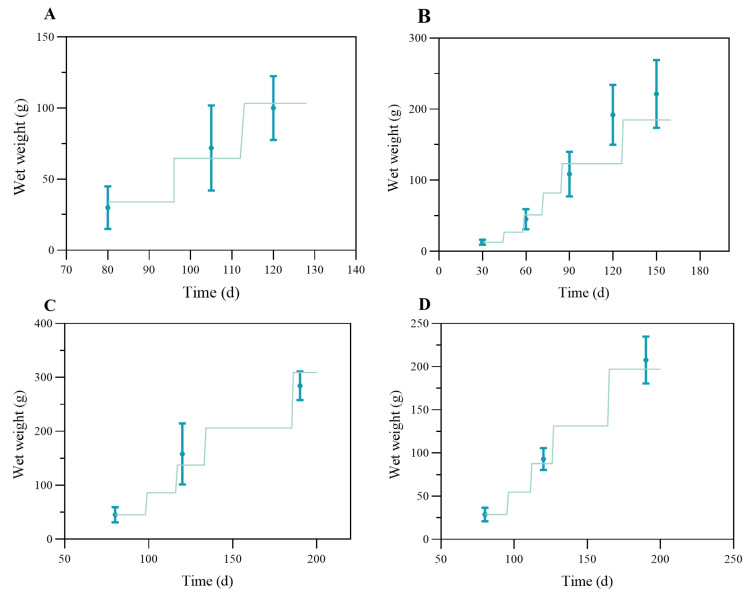
Comparison of the observed (blue dot with error bar) and simulated data (green lines) of wet weight of the swimming crab for different groups in Table 2. With panel (**A**) for dataset A, panel (**B**) for dataset B, panel (**C**) for dataset C, and panel (**D**) for dataset D.

The model results were validated against different observed datasets summarized in Table 1. The results were shown in Figure 7, with different panels representing each dataset. The model had relatively good results for datasets A (R^2^ = 0.977) and D (R^2^ = 0.997). For dataset A, the actual simulation lasted for 50 days; the crab went through two molts and grew from 34 g to about 100 g in total wet weight. For dataset D, the simulated crabs grew from 29 g to more than 200 g in total wet weight, with four molts over 120 days of the culture period. The simulated results for the above datasets at each node sampled for the survey had a good fit to the measured values within a reasonable uncertainty. For dataset B (R^2^ = 0.798), the model performance was relatively average, underestimating the crab growth in the late stage under a higher ambient temperature in the pond (Figure 7B). Within 130 days of model time, the crabs went through five molts and grew from 13 g to 185 g in total wet weight. For dataset C (Figure 7C), where larger crabs (initial $W_{w}$ = 45 g) were cultured under similar forcing to that of dataset D (Figure 7D), the model slightly underestimated growth between days 120 and 140 and overestimated weights near harvest (~309 g), yet it maintained strong agreement overall (R^2^ = 0.963).

**Figure 8:**
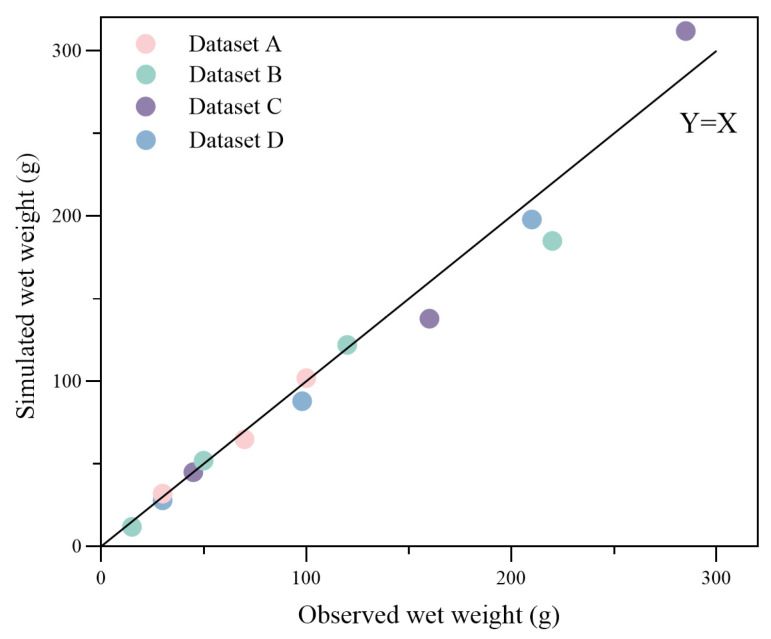
qq-plot of observed and simulated data for wet weight at the observed time. Dots with different colors represent different datasets noted in the legend. The root-mean-square error (with initial values excluded) between the observed and modeled wet weight is 27.46 g.

A quantile–quantile plot was made for the simulated and observed growth data, together with the linear equation, Y = X, as shown in Figure 8. The root-mean-square error (with initial values excluded) between the observed and modeled total wet weight was 27.46 g, with the most substantial standard deviation being 68.73 g, which is about 35.7% of the observed value from group B.

**Figure 9:**
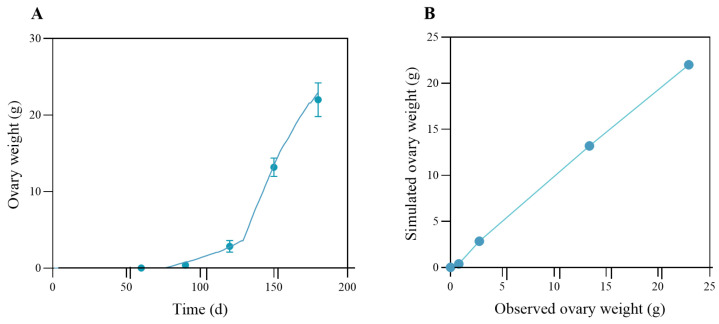
Observed and simulated data for ovary mass. In panel (**A**), the blue dots represent the measured ovary mass, and the solid cyan line represents the modeled ovary mass. In panel (**B**), the qq-plot compares observed and modeled ovary mass data for the swimming crab. The line Y = X represents the line of equality, indicating where the observed values would match the modeled values.

The ovarian simulation results, together with the quantile–quantile plot, are shown in Figure 9A,B. As described, the ovary developed slowly before day ~86 and grew to around 0.8 g before the puberty molt. After the puberty molt, the ovary grew rapidly to more than 20 g around day 180. The model demonstrates a high degree of concordance with the measured data (R^2^ = 0.997; RMSE = 0.48 g).

Statistics of the model performance for growth and ovary simulation are summarized in Table 3. Generally, the model performed reasonably in simulating the individual growth of *P. trituberculatus*. The averaged R^2^ value for the four datasets is 0.93; the averaged Theil’s inequality coefficient and model efficiency are U = 0.116 and ME = 0.94, respectively. Excluding dataset B, the model’s performance metrics improve, indicating that the model performs better under lower environmental temperature conditions. Through iterative calibration, the stage-specific κ-values were finalized as 1.0 (pre-stage VII), 0.9 (stages VIII-X), and 0.2 (post-stage X), consistent with physiological observations.

**Figure 10:**
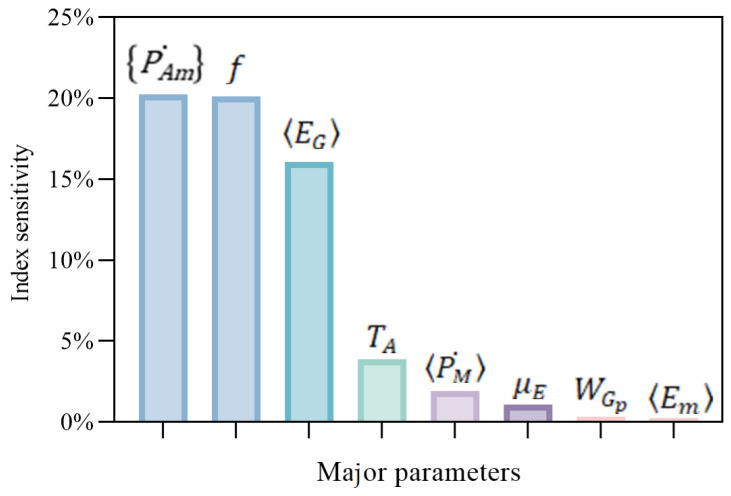
Sensitivity index for parameter variation in the model. This figure illustrates the impact of parameter variations on the model output, specifically, $W_{C}$. The *y*-axis represents the sensitivity index, indicating the relative change in WC due to variations in the corresponding parameters shown on the *x*-axis.

The results of the sensitivity analysis for the major parameters are shown in Figure 10, which revealed that the indicators related to feeding and energy assimilation ($\left\{\dot{P_{Am}}\right\}$ and *f*) had the highest sensitivity index, above 20%. This was followed by the specific energy for structural growth, $\langle E_{G}\rangle$, with an index of 16.06%, and the maximum reserve density, $\langle E_{m}\rangle$, was the least sensitive, with an index of 0.24%. The results are basically consistent with our perception that the feeding parts were the most important in the growth simulation of all of the organisms.

Supplementary Material Table S2 summarizes the DEB model parameters for *P. trituberculatus*, including κ, which were obtained from physiological experiments or literature sources.

## 4. Discussion

### 4.1. Validity of the DEB Model Parameters

Parameterization lies at the core of the DEB model, determining the extent to which model predictions reflect biological reality [12,20,28]. For *P. trituberculatus*, public information and data are limited, particularly regarding assimilation, maintenance, and reproductive costs. Therefore, we designed several physiological experiments based on the principles of the DEB theory to estimate the model parameters. Within an individual-based DEB framework and under the assumption of sufficient feeding (typical of intensive pond aquaculture), energy acquisition is represented as a function of ingestion and assimilation—with the canonical scaling to body-surface area at approximately two-thirds the power of biovolume—reflecting the physiological capacity of digestive and absorptive tissues [5]. In crustaceans, however, exoskeletal constraints render morphological size effectively fixed between molts; as a result, parameterizing ingestion and assimilation by morphological surface area remains contentious [13]. Therefore, in this study, we attempted to scale assimilation with structural weight to the 2/3 power, arguing that structural weight is more aligned with the functional scale of absorption-related tissues and exhibits higher temporal stability throughout the molting cycle. As shown in Figure 5, the results for assimilated energy and estimated structural weight generally support this hypothesis.

In the DEB theory, the energy allocation parameter, κ, governs the partitioning of mobilized reserves between somatic demands (maintenance and growth) and the reproductive channel (including maturity maintenance). Reported values of κ span a broad range across aquatic taxa: the κ is 0.94 for the Chinese shrimp, *F. chinensis* [11]; 0.9 for the abalone, *Haliotis discus hannai* [28]; 0.62 for the scallop, *P. yessoensis* [29]; 0.45 for Pacific oyster, *C. gigas* [30]; and 0.56 for Yellow Croaker, *L. polyactis* [8]. Furthermore, in most of these DEB models, κ is treated as a constant throughout ontogeny. However, the development of the swimming crab, *P. trituberculatus*, exhibits a distinct biphasic pattern—after the puberty molt, the rate of gonadal development increases significantly [14]. Thus, a constant κ value obscures the ontogenetic changes in energy allocation between somatic and reproductive investments. To capture this ontogenetic transition, we implemented segmented κ values representing three energetic stages. This segmentation strategy successfully reproduced the observed periodic growth pattern of *P. trituberculatus*, indicating that stage-dependent κ can effectively represent the dynamic energy trade-offs during molting and maturation and be treated as a state-dependent strategy [31].

### 4.2. Mechanistic Representation of the Molting Process

Molting represents one of the most prominent and energy-intensive events in the life cycle of crustaceans, governing individual growth and reproductive preparation [32,33]. The α-based molting mechanism developed here reframes molting as a state-dependent energetic threshold phenomenon. This model adheres to the conceptual framework proposed by Talbot et al. for *L. depurator* [13], where the α ratio of $W_{C}$ (organic matter) to $W_{W}$ (exoskeleton space) serves as the molt-initiating signal. Molting is triggered once ratio α exceeds a critical threshold, indicating that internal reserves have reached a level incompatible with the constraints imposed by the current exoskeleton. Through this relationship, the association between internal energetic status and morphological constraints is quantified.

For the swimming crab, *P. trituberculatus*, the number of molts exceeds 10 over its entire lifespan, with post-molt body weight gain decreasing as the number of molts increases. Observational data reveal that $\alpha_{post}$ remains nearly constant across different size classes; in contrast, $\alpha_{pre}$ exhibits distinct variations across different molting stages. During early growth stages, higher $\alpha_{pre}$ reflects a faster growth rate and shorter molt intervals, likely due to the need for frequent molting in early life to rapidly increase body length and weight, thereby enhancing survival rates [34]. By comparison, $\alpha_{pre}$ gradually stabilizes in late growth stages, which is likely associated with a shift in the energy allocation strategy of swimming crabs—a gradual redirection of energy utilization toward reproductive development rather than sustained growth. Thus, the accurate determination of stage-specific $\alpha_{pre}$ thresholds is critical for predicting intermolt periods and molt increments. Furthermore, quantification of $\alpha_{pre}$ and $\alpha_{post}$ across diverse species could yield a comparative molt index analogous to metabolic scaling exponents [35,36], a framework that would connect energetic storage strategies to life-history diversity in crustaceans [37,38].

### 4.3. Model Performance on Maturity Prediction

According to the κ-rule, the κ fraction of mobilized reserve is allocated first to somatic maintenance, with the remainder supporting structural growth; the (1 − κ) fraction is allocated first to maturity maintenance, with the remainder supporting development or (in adults) reproduction. Therefore, the instantaneous allocations to growth and reproduction are variable over time. However, this assumption is difficult to reconcile with the inherently cyclical nature of crustacean growth and development. This inconsistency is particularly pronounced in their reproductive biology: gonadal development is periodic, highly synchronized with the molt cycle, and concentrated in the late intermolt stages. Many previous individual-based crab models have overlooked this periodicity in gonadal development. To address this discrepancy, we extended the DEB framework by introducing a demand-driven gonad-loading feedback mechanism and used segmented κ values to simulate the growth cycle of the swimming crab.

The successful simulation of ovarian growth and maturation timing validates this dynamic feedback mechanism. Specifically, the model effectively reproduces empirical observations of ovarian growth trajectories across multiple reproductive cycles, demonstrating its ability to capture the inherent periodicity of energy allocation in the swimming crab. Meanwhile, this study demonstrates that the demand-driven gonad-loading feedback mechanism, centered on the reproductive reserve to gonadal demand axis, can serve as a viable solution applicable to diverse taxa and reproductive strategies.

For aquaculture, these insights hold direct implications. First, by clarifying the interrelationship between molting and reproduction, the onset of sexual maturity can be accurately predicted. Second, it provides a quantitative basis for determining feeding regimens, harvest schedules, and female crab conditioning. This further enables the estimation of spawning timing, thereby improving production efficiency.

## 5. Conclusions

This study advances the application of the DEB model in crustacean physiology by embedding discontinuous molting and stage-specific energy allocation into a unified mechanistic framework. This approach not only enhances the accuracy of growth predictions but also elucidates the fundamental energetic trade-offs underpinning the life-history strategies of decapod crustaceans. The α-based molting mechanism quantitatively links the internal energetic reserve status of crustaceans to the morphological constraints of their exoskeleton; meanwhile, the stage-specific segmented κ parameters and the gonadal development feedback mechanism capture the trade-off strategy of shifting energy allocation from somatic growth to reproductive investment.

From a broader perspective, numerous commercially valuable crustaceans, such as the mud crab, *Scylla paramamosain*; the Chinese mitten crab, *Eriocheir sinensis*; and *Charybdis japonica*, exhibit analogous molt-dependent growth and reproduction patterns [39,40,41]. Applying the current modeling strategy to these species can facilitate cross-species comparisons of energetic efficiency and adaptive life-history strategies. Coupling this individual-based framework to population and ecosystem models will support ecosystem-based aquaculture by linking individual energetics to population dynamics and trophic cycling and contribute to more efficient aquaculture management and lower environmental impacts.

## Figures and Tables

**Table 1:** Growth data used for model validation.

No.	Sample Size	Initial Wet Weight (g)	Culture Location
A	1140	34.00	Weifang, Shandong
B	120	12.88	Ningbo, Zhejiang
C	22	45.18	Weifang, Shandong
D	18	28.79	Weifang, Shandong

**Table 2:** Selection of $\alpha_{\mathrm{pre}}$ in different growth stages.

Stage	I	II	III	IV	V	VI	VII	VIII	IX	X	XI *
$\alpha_{pre}$	0.27	0.44	0.30	0.30	0.30	0.26	0.21	0.19	0.16	0.15	0.15

* XI is puberty molt.

**Table 3:** Statistics for model evaluation with observation data.

Statistic	Sample Size	$R^{2}\;\left[0,\;1\right]$	$U\;\left[0,\;\infty \right]$	$\mathrm{ME}\;\left[0,\;1\right]$
A	1140	0.977	0.0556	0.977
B	120	0.798	0.257	0.811
C	22	0.963	0.0985	0.963
D	18	0.997	0.0520	0.991
E (ovary)	30	0.997	0.0419	0.997

## Data Availability

The data can be shared upon request.

## Supplementary Materials

The following supporting information can be downloaded at https://www.mdpi.com/article/10.3390/biology14121682/s1: S1. Supplementary Table. Table S1: State variables, model equations, and biological functions; Table S2: Parameters for the DEB model. S2. Laboratory experiments. S3. Model evaluation metrics.
