# Supplementary material for: A Dynamic Energy Budget Model for the Non-Continuous and Biphasic Growth of the Pond-Cultured Swimming Crab, Portunus trituberculatus
Source: Biology (Basel). 2025 Nov 26;14(12):1682. doi: 10.3390/biology14121682 (PMC12730681; doi:10.3390/biology14121682)
Supplement: Supplementary file 1 [file biology-14-01682-s001.zip › biology-3996200-supplementary.pdf]

# A Dynamic Energy Budget Model for the Non-Continuous and Biphasic Growth of the Pond-Cultured Swimming Crab, *Portunus trituberculatus*

**Yi Jiang [1,†], Fan Lin [1,2,†], Jingyan Zhang [1,2], Ming Bao [1], Baoquan Gao [1,2], Jitao Li [1,2] and Xianliang Meng [1,2,3,*]**

[1] State Key Laboratory of Mariculture Biobreeding and Sustainable Goods, Yellow Sea Fisheries Research Institute, Chinese Academy of Fishery Sciences, Qingdao 266071, China
[2] Laboratory for Marine Fisheries Science and Food Production Processes, Qingdao Marine Science and Technology Center, Qingdao 266237, China
[3] Key Laboratory of Aquatic Genomics, Ministry of Agriculture and Rural Affairs, Yellow Sea Fisheries Research Institute, Chinese Academy of Fishery Sciences, Qingdao 266071, China
* Correspondence: xlmeng@ysfri.ac.cn; Tel./Fax: +86-532-8583-6605
† These authors contributed equally to this work.

## Content

S1. Supplementary Table ........ 2
Table S1.1 State variables, model equations and biological functions ........ 2
Table S1.2 Parameters for the DEB model ........ 3
S2. Laboratory experiments ........ 4
S2.1 Animals and holding system ........ 4
S2.2. Oxygen consumption across temperatures and Arrhenius temperature ........ 5
S2.3. Starvation experiment ........ 6
S2.4. Feeding experiment ........ 8
S2.5. Ovarian energetics and molt thresholds experiment ........ 9
S3. Model evaluation metrics ........ 11
S3.1. R-square ........ 11
S3.2. Model efficiency ........ 12
S3.3. Thiel's inequality coefficient ........ 12
S3.4. Sensitivity analyses ........ 12
References ........ 13

## S1. Supplementary Table

Table S1. State variables, model equations, and biological functions.

| Symbol | Description | Equation/Formula | Unit |
|---|---|---|---|
| | | **Model equations** | |
| $E$ | Reserves | $\frac{dE}{dt} = p_A - p_C$ | J |
| $W_G$ | Structural weight | $\frac{dW_G}{dt} = \frac{\kappa p_C - p_M}{[E_G]}$ | g |
| $B_F$ | Reproduction buffer stock mass | $\frac{dB_F}{dt} = \frac{((1-\kappa)p_C - p_J)}{\langle E_{B(A)O} \rangle}$ | g |
| $W_E$ | Reserve weight | $\frac{E}{\mu_E}$ | g |
| $m_E$ | Density of general reserves | $\frac{W_E}{W_G}$ | - |
| $B_G$ | Reproductive matter stock mass | $y_G k_f m_{RE} m_G (m_{Gm} - m_G) W_G$ | g |
| $m_{RE}$ | Density of reproductive reserves | $\frac{dm_{RE}}{dt} = \frac{k_{RG} B_F}{W_G} - j_V m_{RE} - \frac{k_{RE} B_G}{W_G y_G}$ | - |
| $m_G$ | Density of reproductive matter | $\frac{dm_G}{dt} = \frac{k_{RE} B_G}{W_G} - j_V m_G$ | - |
| $W_{OC}$ | Ovarian carbon weight | $m_G W_G$ | g |
| $W_O$ | Ovarian weight | $\kappa_{ov} W_{OC}$ | g |
| | | **Biological function** | |
| $f$ | Function response | $X/\,(X + X_K)$ | - |
| $k(T)$ | Temperature dependence | $k_0 \exp\,(\frac{T_A}{T_0} - \frac{T_A}{T})$ | - |
| $p_A$ | Assimilation rate | $\mathrm{k}(T)\, f\{\dot{p}_{Am}\} W_G^{2/3}$ | $J \cdot d^{-1}$ |
| $p_C$ | Catabolic rate | $\mathrm{k}(T)\ \frac{\mathrm{f}[\mathrm{E_m}][\mathrm{E_G}]}{\kappa \cdot \mathrm{f}[\mathrm{E_m}] + [\mathrm{E_G}]} \left( \frac{\{\dot{p}_{Am}\} W_G^{2/3}}{[E_m]} + \frac{\langle \dot{P_M} \rangle W_G}{[E_G]} \right)$ | $J \cdot d^{-1}$ |
| $p_M$ | Structural maintenance rate | $\langle \dot{P_M} \rangle W_G\ k(T)$ | $J \cdot d^{-1}$ |
| $p_J$ | Maturity | $\mathrm{k}(T)\ \min(W_G, W_{G_p}) \langle \dot{P_M} \rangle^{\frac{1-\kappa}{\kappa}}$ | $J \cdot d^{-1}$ |

| | | | |
|---|---|---|---|
| | maintenance rate | | |
| $\dot{j}_V$ | Specific growth rate | $\frac{1}{W_G}\frac{dW_G}{dt}$ | $d^{-1}$ |
| | **Molt** | | |
| $W_C$ | Carbon weight | $W_G + W_E$ | g |
| $W_{w[m*]}$ | Body wet weight (change every molt） | $\frac{\alpha_{pre} \cdot W_{w[m*-1]}}{\alpha_{post}}$ | g |
| $TW_w$ | Total wet weight | $W_{w[m*]} + W_O$ | |
| $\alpha$ | The molt coefficient | $W_C / W_W$ | - |

Notes: m in wet weight represents the value at the $m^{th}$ molt.

Table S2. Parameters for the DEB model.

| Symbol | Definition | Value | unit |
|---|---|---|---|
| $T_0$ | Reference temperature | 298.15 | K |
| $T_A$ | Arrhenius temperature | 5482 | K |
| $[E_G]$ | Weight-specific costs for structure | 51180 | $J \cdot g^{-1}$ |
| $[E_m]$ | Maximum reserve density | 3860 | $J \cdot g^{-1}$ |
| $[\dot{P}_M]$ | Weight-specific maintenance rate | 310 | $J \cdot g^{-1}\ d^{-1}$ |
| $\mu_E$ | Energy content of reserves | 18750 | $J \cdot g^{-1}$ |
| $[E_{BO}]$ | Weight-specific costs for ovary before mating | 36690 | $J \cdot g^{-1}$ |
| $[E_{AO}]$ | Weight-specific costs for ovary after mating | 66200 | $J \cdot g^{-1}$ |
| $AE$ | Assimilation efficiency | 0.95 | - |
| $\{\dot{p}_{Am}\}$ | Maximum structural weight $^{2/3}$ specific assimilation rate | 4590 | $J \cdot g^{-2/3} \cdot d^{-1}$ |
| $\kappa$ | Fraction of catabolic flux to growth and maintenance before ovary development | 1 | - |
| $\kappa_{BO}$ | Fraction of catabolic flux to growth and maintenance after ovary development and before | 0.90 | - |

| | | | |
|---|---|---|---|
| | mating | | |
| $\kappa_{AO}$ | Fraction of catabolic flux to growth and maintenance after mating | 0.20 | - |
| $W_{G_p}$ | Structural body weight at puberty | 6.78 | g |
| $m_{Gm}$ | Maximum density of reproductive matter | 0.074 | - |
| $k_f$ | The conversion coefficient of reproductive material structural weight | 10.0 | - |
| $y_G$ | Conversion efficiency reproductive reserve to reproductive matter | 1 | - |
| $k_{RG}$ | Reproductive matter turnover rate | 1 | $d^{-1}$ |
| $k_{RE}$ | Reproductive reserve turnover rate | 1 | $d^{-1}$ |
| $\kappa_{ov}$ | Fraction of reproductive matter in ovaries | 0.085 | |
| $\alpha_{post}$ | Carbon weight /wet weight ratio minimum | 0.1 | - |
| $\alpha_{pre}$ | Carbon weight /wet weight ratio maximum | 0.15~0.44 | - |

Note: The parameter sources mainly include physiological experiments and model debugging (calibration).

## S2. Laboratory experiments

Several physiological experiments were carried out for parameter estimation of the DEB model, including the measurement of oxygen consumption under different temperatures, feeding, starvation, and molting conditions and the ovarian development cost. The index of testis is very low (~0.2%), and the energy cost for testicular development seems negligible [1]. In addition, female crabs with fully developed ovaries have much higher economic value than male ones. Therefore, only the ovarian development cost was investigated in this study.

### S2.1 Animals and holding system

Parameterization of the model requires comprehensive information on the

physiology of the crab. Most physiological experiments were conducted in a recirculating system with live crabs obtained from the culture ponds in Weifang, China (N:36°42′10.44″, E:119°3′14.4″).

A recirculating system with 30 separate tanks (20 L) was operated under optimum conditions for the swimming crab. Before each experiment, the crabs were temporarily reared in the recirculating system for 7 days for acclimation. In the recirculating system, the water temperature in each tank was around 25.0 ± 1.0 °C, the salinity was 30.3 ± 0.6, pH was 7.6 ± 0.2, the ammonia nitrogen concentration was below 0.03 $mg·L^{-1}$, and the dissolved oxygen concentration was 6.9 ± 0.2 $mg·L^{-1}$. During acclimation, all the crabs were fed with fresh fish once a day, and the feces and waste feed were removed before the next feeding.

S2.2. Oxygen consumption across temperatures and Arrhenius temperature

The Van't Hoff–Arrhenius equation is often used to describe the temperature dependence of physiological rates in the DEB theory. The oxygen consumption rates (ROCs) at different temperatures were used to estimate the Arrhenius temperature (TA), referring to a previous methodology [2]. In total, five datasets from different wet weight groups were used, including three groups of small crabs (1.17 ± 0.03 g; 57.57 ± 1.16 g; 78.19 ± 7.76 g) from the published data [3-4], and two groups of large crabs (100.88 ± 11.22 g; 174.97 ± 25.72 g) from the physiological experiments in this study. The Arrhenius temperature can be estimated from the linear regression between the Napierian logarithm of the oxygen consumption rate and the reciprocal of the absolute temperature:

$$ln\, R_{OC} = a \times T^{-1} + b \ldots\ldots (1)$$

where the absolute value of slope a represents TA, and b represents the intercept. During the oxygen consumption experiment, the crabs (five individuals for each weight group) were transported from the recirculating system to separate containers with a volume of 10 L. The water temperature in each container was increased or decreased gradually (±1 °C per day) from the rear temperature to the designed temperatures (7, 13, 19, 25, and 31°C). After being maintained at the experimental temperatures for 3 days, the ROC of each crab was measured with a hydrostatic method [4]. For each temperature, a control experiment without a crab was set up to measure the background oxygen consumption of bacteria or other likely organisms, and this background value was subtracted from the experimental group. Dissolved oxygen concentrations in each container were determined using the iodometric method [5], and ROC was calculated from the decrease in dissolved oxygen concentration over 2 h. To better estimate TA for *P. trituberculatus*, datasets from previous studies [3-4] were analyzed as supplements to the physiological experiment data.

S2.3. Starvation experiment

Starvation experiments were carried out in the same recirculating system for temporary rearing, totaling 36 crabs with barely developed ovaries (125.74 ± 32.00 g), which were measured to estimate energetic parameters (e.g., maximum reserve density, $[E_m]$, weight-specific maintenance rate, $[\dot{P}_M]$, and energy for unit structural mass, $[E_G]$) under the optimum temperature of 25 °C. When the experiment started, six crabs were randomly collected every other day and processed to measure the total

wet weight ($W_W$), carbon weight ($W_C$), oxygen consumption rate (ROC), and reserve energy (E). $W_W$ was measured with the XS802S balance (Mettler Toledo, Switzerland) directly after carefully wiping surface water off the crab with absorbent paper. When the measurement of $W_W$ was finished, the crabs were placed in a 60 °C oven to dry the moisture until the mass remained unchanged; the dried body flesh weight of the crabs was then weighed after cooling for 3 h. After this, the dried crabs were ashed in a muffle furnace at 550°C for 4 h and weighed, and the $W_C$ values of the crabs were obtained by deducting the ash weight from the dried body flesh weight. The gross energy of the samples was determined with a PARR1281 oxygen bomb calorimeter (PARR Instrument, USA). The experiment was terminated after 10 days when the reserves were mostly used up by the crabs. Referring to Ren and Schiel [6], the energy of reserves is defined as the component of carbon weight that can be lost during starvation, and the maximum reserve density is calculated as

$$[E_m] = \frac{E_0 - E_1}{W_1} \ldots\ldots (2)$$

The energy content of reserves is calculated as

$$\mu_E = \frac{E_0 - E_1}{W_0 - W_1} \ldots\ldots (3)$$

where $E_0$ and $E_1$ represent the energy of the carbon weight at the beginning and end stages of the starvation period, and $W_1$ is the carbon weight at the end of the experiment, which is assumed to be the structure weight, $W_G$, of the crabs at the moment. When both the carbon weight and oxygen consumption rate measured remained approximately constant during starvation, the reserves were considered emptied [6]; the ratio of structure weight to carbon weight can be obtained as $\beta = W_1/W_0$ for crabs under abundant food conditions, where $W_0$ is the initial carbon weight.

The structural weight-specific maintenance rate can be estimated as

$$[\dot{p}_M] = \frac{E_{O_2} \times R_{OC}}{W_1} \dots\dots (4)$$

where $E_{O_2}$ is the heat of combustion of oxygen (15.496 J·mg$^{-1}$), $R_{OC}$ is the stable oxygen consumption rate at the end of the experiment, and $W_1$ is the same as above. The weight-specific energy cost for structure growth, $[E_G]$ can be estimated as

$$[E_G] = \frac{E_1}{W_1 \times T_r} \dots\dots (5)$$

where $E_1$ and $W_1$ are the same definition as above, and $T_r$ is the growth conversion efficiency, with a value of 40% [2].

S2.4. Feeding experiment

The feeding experiment was carried out to estimate the assimilation-related parameters (e.g., assimilation efficiency and maximum-surface-area-specific assimilation rate, $\{\dot{p}_{Am}\}$). Eight crabs (33.52 ± 27.23 g) were used to correlate energy assimilation and body sizes. Similar to the starvation experiment, each crab was placed in a separate tank under the optimum rearing conditions [k($T$) = 1] in the recirculating system. Before feeding, the crabs were starved for 24 hours to empty the intestines, and the wet weights were measured. The feeding experiment started at 17:00 with sufficient feed supplied and weighed in each tank. Three control tanks were set without crabs to estimate errors caused by the dissolution of feed in water. The crabs were free-fed for 24 h, and after that, the uneaten feed was collected and weighed. The feces of the crabs were also collected instantly in each tank; the wet weight and carbon weight of the crabs were measured until no new feces were produced, using the same method as the starvation experiment. The ingested feed is calculated as the difference between the supplied feed and the sum of dissolved and uneaten feed. The energy level for unit feed and feces was measured with a

PARR1281 oxygen bomb calorimeter (PARR Instrument, USA), thus transforming the assimilated feed into energy. The energy assimilation rate ($AE$) can be calculated as

$$AE = \frac{(E_x - E_F)}{E_x} \times 100\% \dots\dots (6)$$

where $E_x$ is the energy of the ingested feed, and $E_F$ represents the energy of feces. With the ingestion rate and measured carbon weight, the maximum-structural-area-specific assimilation rate ($J \cdot g^{-2/3} \cdot d^{-1}$) can be estimated as

$$\{\dot{p}_{Am}\} = \frac{AE \times E_x}{(\beta W_C)^{2/3}} \dots\dots (7)$$

in which $W_G$ is the structure weight of the crabs under the assumption of sufficient feed supply ($f$=1). The structure weight is estimated from the carbon weight with ratio $\beta$, under the assumption that the reserve of the ectotherm is always at equilibrium with a sufficient food supply [2], and the ratio of structure weight to carbon weight is constant for all immature crabs.

S2.5. Ovarian energetics and molt thresholds experiment

To compare the differences in ovarian development of *P.trituberculatus* before and after reproductive molting, we selected female crabs at two distinct developmental stages: pre-reproductive molting (weight range, 80-100 grams; mean wet weight, 90±5 grams; n=10) and post-reproductive molting (weight range, 100-125 grams; mean wet weight, 110±10 grams; n=10). The crabs were anesthetized using an ice-water mixture, and their wet weights were recorded. The ovaries were meticulously excised and immediately weighed to an accuracy of 0.01 grams. Subsequently, the carbon content and total energy levels of both the body and ovaries were determined following the methodology established in our previous starvation

experiment. The energy per unit carbon mass of ovary growth ($J \cdot g^{-1}$) before and after the reproductive molt was then calculated for the crabs as

$$E_{B(A)O} = \frac{E_{B(A)O}}{W_{CB(A)} \times T_r} \ldots \ldots (8)$$

where $E_{B(A)O}$ represents the ovary energy for crabs before (after) the puberty molt, $W_{CB(A)}$ represents the carbon mass of the ovary at the same stage, and $T_r$ is the growth conversion rate.

Simultaneously, the threshold density of reproductive material for the swimming crab was estimated as

$$m_{Gm} = \frac{W_{CM}}{(1-\beta)W_{GM}} \ldots \ldots (9)$$

where $W_{CM}$ represents the ovarian carbon weight of fully mature female crabs, and $W_{GM}$ represents the carbon weight of female crabs with fully developed ovaries.

Simulation of molting requires data for the critical ratio of $\alpha_{pre}$ and $\alpha_{post}$. From June to October, healthy female *P. trituberculatus* crabs were randomly selected and placed in individual transparent tanks under the same experimental conditions as in the previous experiments. During the experiment, the molting process of each crab was recorded until the new exoskeleton fully hardened. Subsequently, female crabs that had just completed molting (weight range, 12.03-167.84 grams; n=16) were selected. Their wet weight and carbon content were measured using the same methods as in the starvation experiment to determine the critical value $\alpha_{post.}$ Our observations of the molt cycles of *P. trituberculatus* revealed variation in the relative weight gain rates after different cycles, which means that the $\alpha_{pre}$ values of different molting cycles were distinct. In contrast, the $\alpha_{post}$ in different molting cycles was basically the same. Data on the molt of *P. trituberculatus* published by Gao et al. were also employed for the analysis of $\alpha_{pre}$ and $\alpha_{post}$ [7]. The carbon weight ratio after molting is

calculated as

$$\alpha_{post} = \frac{W_C}{W_w} \dots\dots (10)$$

where $W_C$ and $W_w$ are the values measured just after each molt. The critical ratio before molt is given as

$$\alpha_{pre} = \frac{W_w}{W_{wB}} \times \alpha_{post} \dots\dots (11)$$

where $W_w$ represents the weight mass after the molt, and $W_{wB}$ is the wet mass before the molt.

## S3. Model evaluation metrics and sensitivity analyses

We employed several datasets to validate the model, including data from previous publications related to the growth of *P. trituberculatus* [8-9] and growth data recorded from crab culture ponds, as summarized in Table 1. Additional data for gonadal growth validation were taken from Jia [10].

The performance of the DEB model for *P. trituberculatus* was evaluated by calculating the coefficient of determination ($R^2$) for simulated and observed values, the model efficiency (ME), and Thiel's inequality coefficient (U).

### S3.1. R-square

$R^2$ indicates the variation explained by the model proportion: $R^2 = 1$ refers to a perfect fit of the model data and observation, while models with a poorer fit have lower $R^2$ values. The coefficient of determination is calculated as

$$R^2 = 1 - \frac{\sum_i (O_i - S_i)^2}{\sum_i (O_i - \bar{O})^2} \dots\dots (12)$$

where $i$ represents the number of observations, $O_i$ represents the observation value, $\bar{O}$ is the observation mean, and $S_i$ represents the corresponding simulation results.

S3.2. Model efficiency

The model efficiency is calculated as

$$ME = 1 - \frac{\sum_{i=1}^{n} i_2^D}{\sum_{i=1}^{n}(O_i - \bar{S})^2} \ldots \ldots (13)$$

where $D_i = O_i - S_i$, and $\bar{S}$ represents the averaged simulation values. *ME* equals 1, indicating a perfect fit, and *ME* equals 0, indicating that the model is not better than a simple average of the estimates, with a negative value indicating poor model performance.

S3.3. Thiel's inequality coefficient

Thiel's inequality coefficient can take a value of 0 or greater and is calculated as

$$U = \sqrt{\frac{\sum_{i=1}^{n} i_2^D}{\sum_{i=1}^{n} i_2^O}} \ldots \ldots (13)$$

If U equals 0, the model simulates perfectly; if U equals 1, the model produces estimates that are not better than assuming constant variables; and if U is greater than 1, the model has unacceptable predictive power.

The statistics of the model performance for growth and ovary simulation are summarized in **Error! Reference source not found.**. Generally, the model performed reasonably in simulating the individual growth of *P. trituberculatus*. The averaged $R^2$ value for the four datasets is 0.93; the averaged Thiel's inequality coefficient and model efficiency are U = 0.116 and ME=0.94, respectively.

S3.4. Sensitivity analyses

Sensitivity analyses were applied to the major model parameters according to Majkowski [11], with a correction of ± 10% for each parameter, and the result of each test was analyzed using the sensitivity index to calculate the obtained percentage

variation in $W_C$. The equation for the sensitivity index calculation is given as

$$SI = \frac{1}{n}\sum_{t=1}^{n}\frac{\left|W_{t_1^C} - W_{t_0^C}\right|}{W_{t_0^C}} \times 100\% \ldots\ldots (14)$$

where *n* is the number of simulated days, $W_{t_o^C}$ is the carbon mass predicted with the standard simulation at each time step, and $W_{t_n^C}$ is the carbon mass predicted with a new parameter.

# References


1. Che, J., Liu, M., Hou, W., Dong, Z., Yang, S., Cheng, Y., Wu, X., 2019. Growth and gonadal development of pond-reared male swimming crab, *Portunus trituberculatus*. *Chinese Journal of Zoology*, 54(3), 347-361.
2. Van der Meer, J., 2006. An introduction to Dynamic Energy Budget (DEB) models with special emphasis on parameter estimation. *Journal of Sea Research* 56(2), 85-102.
3. Dai, C., Wang, F., Fang, Z., Dong, S., 2014. Effects of temperature on the respiratory metabolism and activities of related enzymes of swimming crab *Portunus trituberculatus*. *Progress in Fishery Sciences* 35(02), 90-96.
4. Lu, Y., 2015. Basic research about effects of temperature on metabolic physiology of the swimming crab Portunus trituberculatus. Doctoral dissertation, Ocean University of China.
5. Lei, Y., 2006. Chemistry Experiments on Aquaculture Water Environment. Beijing: China Agriculture Press.
6. Ren, J.S., and Schiel, D.R., 2008. A dynamic energy budget model: parameterisation and application to the Pacific oyster *Crassostrea gigas* in New Zealand waters. *Journal of Experimental Marine Biology and Ecology* 361(1), 42-48.
7. Gao, T., Wang, Y., Bao, X., Ren, Z., Mu, C., Wang, C., 2016. Study on the characteristics of molting and growth of *Portunus trituberculatus* cultured in single individual basket. Journal of Biology 33(03),41-46.

8. Liu, L., Li, J., Gao,B.,Liu, P., Dai, F., Pan, L., 2009. Correlation of growth traits of *Portunus trituberculatus* at the different ages and its impact on body weight. *Journal of Fisheries of China* 33(06),964-971.
9. Wang, Y., Chen, C., Bao, X., Mu, C., Song, W., Li, R., Peng, X., Wang, C., 2014. Morphometric growth of *Portunus trituberculatus*" Zhongning No. 1". *Journal of Fisheries of China* 38(02),183-192.
10. Jia, L., 2008. Preliminary Study on the ovary development of the crab *Portunus trituberculatus*. Master's dissertation, Ocean University of China.
11. Majkowski, J.,1982. "Usefulness and applicability of sensitivity analysis in a multispecies approach to fisheries management", in: Theory and management of tropical fisheries. *ICLARM Conf. Proc*. 9, 149-165.
